# Effect of the vision suppression on the graphomotor gesture in school aged children typically developed and with handwriting disorders

**DOI:** 10.1192/j.eurpsy.2022.1939

**Published:** 2022-09-01

**Authors:** C. Lopez, L. Vaivre-Douret

**Affiliations:** 1 National Institute of Health and Medical Research (INSERM UMR 1018-CESP), University of Paris-Saclay, UVSQ, Faculty Of Medicine, Villejuif, France; 2 Faculty of Health, Université de Paris, Department Of Medicine, Paris, France; 3 Imagine Institute Necker hospital, Department Of Endocrinology, Paris, France; 4 University Institute of France, (iuf ), Paris, France; 5 Necker hospital AP-HP .Centre, Child Psychiatry, Paris, France

**Keywords:** vision suppression, prescriptural task, proprioception, Graphomotor gesture

## Abstract

**Introduction:**

The knowledge aboutthe integration of letter motor programs during learning to write support the idea of an interdependence of visual and kinesthetic controls to direct the strokes.

**Objectives:**

The objective of our study is to analyze the effect of the vision suppression both on the postural-gestural organization and on the spatial/temporal/kinematic parameters in a prescriptural task.

**Methods:**

35 school aged children with handwriting disorders (HD group) aged 6-11 years and 35 matched typical children were included in the study. They performed a prescriptural task of copying a cycloid line of loops, carried out under two conditions, with open eyes versus closed eyes. Postural-gestural measures were recorded with two video cameras allowing 2D reconstruction of the gesture. Spatial/temporal/kinematic measures were recorded with a digital pen.

**Results:**

The HD group showed a significantly poorer postural control and an improvement in the spatial/temporal/kinematic parameters of the loops when they closed their eyes compared to eyes open. In typical group, the postural-gestural organization became significantly more mature but with no significant influence on the spatial/temporal/kinematic parameters of the loops.

**Conclusions:**

HDs could be partly explained by a deficit in the processing of proprioceptive/kinesthetic feedback and a disruptive effect of the visual control on the quality of the prescriptural drawings. The ability to direct the strokes would remain dependent on sensory feedbacks, themselves insufficiently efficient, which would lead to difficulties in reaching a proactive control of handwriting. These results should be able to enhance clinical practices and to contribute to clinical decision making processes for handwriting disorders remediation.

**Disclosure:**

No significant relationships.

